# Relationship of Blood and Urinary Manganese Levels with Cognitive Function in Elderly Individuals in the United States by Race/Ethnicity, NHANES 2011–2014

**DOI:** 10.3390/toxics10040191

**Published:** 2022-04-14

**Authors:** Arturo J. Barahona, Zoran Bursac, Emir Veledar, Roberto Lucchini, Kim Tieu, Jason R. Richardson

**Affiliations:** 1 Department of Environmental Health Sciences, Robert Stempel College of Public Health and Social Work, Florida International University, Miami, FL 33199, USA; arbaraho@fiu.edu (A.J.B.); rlucchin@fiu.edu (R.L.); ktieu@fiu.edu (K.T.); 2 Department of Biostatistics, Robert Stempel College of Public Health and Social Work, Florida International University, Miami, FL 33199, USA; zbursac@fiu.edu (Z.B.); emirv@baptisthealth.net (E.V.); 3 Baptist Health South Florida, Miami, FL 33199, USA

**Keywords:** manganese, neurotoxicity, environmental exposure, cognitive function, race, ethnicity, Alzheimer’s disease, CERAD, animal fluency, DSST

## Abstract

Manganese (Mn) is an essential metal with a biphasic relationship with health outcomes. High-level exposure to Mn is associated with manganism, but few data explore the effects of chronic, lower-level Mn on cognitive function in adults. We sought to determine the relationship between blood/urinary manganese levels and cognitive function in elderly individuals using 2011–2014 data from the National Health and Nutrition Examination Survey (NHANES). Weighted multivariate regression models were used to determine correlations, adjusting for several covariates. Blood Mn was inversely associated with the Consortium to Establish a Registry for Alzheimer’s Disease (CERAD) immediate learning of new verbal information (*p*-value = 0.04), but lost significance after adjusting for medical history (*p*-value = 0.09). In addition, blood Mn was inversely associated with Animal Fluency scores after adjusting for all covariates. Urinary Mn was inversely associated with CERAD immediate learning after adjusting for all covariates (*p*-value = 0.01) and inversely associated with the Digit Symbol Substitution Test scores (*p*-value = 0.0002), but lost significance after adjusting for medical history (*p*-value = 0.13). Upon stratifying by race/ethnicity, other Races and Non-Hispanic (NH)-Blacks had significantly higher blood Mn levels when compared to NH-Whites. Collectively, these findings suggest that increased blood and urinary Mn levels are associated with poorer cognitive function in an elderly US population.

## 1. Introduction

Manganese (Mn) is a trace metal with various essential functions in the human body and plays a vital role in the metabolism of glucose and lipids and the synthesis of various proteins [1]. Mn also plays a protective role against oxidative stress; it is a critical component of Mn superoxide dismutase (MnSOD), a reactive oxygen species scavenging enzyme [1]. Furthermore, Mn has been previously linked to sex-related metabolic differences and is essential in reproduction [2]. While there is an average of 0.012 g of Mn in a 70 kg human body, high-level Mn exposure has been linked to neurotoxicity despite the critical functions it plays [3,4]. Acute, high exposure to Mn has been demonstrated to result in manganism, which is clinically manifested as slow and clumsy movements, tremors, difficulty in walking, and facial muscle spasms [5]. Alternatively, chronic exposure has been linked to an extrapyramidal syndrome similar to Parkinson’s disease (PD) [5,6].

Epidemiological studies indicate a possible association between environmental exposure to Mn and cognitive function. It is suggested that the main exposure to Mn is occupational, where it is often inhaled by welders in the construction and agricultural industries [6]. Flynn and Susi found that occupational exposure to Mn is highly correlated to manganism across multiple studies [7]. A cross-sectional study conducted among foundry workers found that more chronic levels of metals such as aluminum, lead, and Mn were correlated with lower levels of cognition and higher stress levels [8]. Additionally, increased blood Mn levels have been associated with decreased cognitive function, as defined by the Auditory Verbal Learning Test in ferroalloy workers [9]. Pathologically, welders with increased chronic Mn exposure have been found to have decreased neural activation when compared to healthy controls, which also correlated with lower executive function, as defined by sorting and word-color tests [10]. 

Several studies have also highlighted associations between chronic environmental exposure to Mn and cognitive function in the general population. Some alternative routes of exposure to Mn may include contaminated water and diet [11,12]. The Environmental Protection Agency (EPA) has set a regulatory standard of 0.05 mg/L in drinking water, while the World Health Organization’s (WHO) standard is 0.08 mg/L [13]. While the WHO standard was set based on the tolerable daily intake for infants, it can be generalized to the whole population, as infants represent the most susceptible subgroup [14]. Nevertheless, experts estimate that over 100 million people remain at risk for Mn toxicity worldwide. This risk is understated as most individuals are also at risk for co-exposure to heavy metals including Pb and As in drinking water [15]. A systemic review conducted by Zoni and Lucchini evaluated data from five countries and found six out of ten articles suggesting adverse effects of Mn on cognitive function, as defined by cognitive, motor, or behavioral changes [16]. Computer modeling data of measured air monitoring paired with cognitive testing suggests chronic environmental exposure to Mn found in the air may result in cognitive deficits in adults [17]. These findings have been validated by a recent study that found an association between high Mn emission and cognitive dysfunction in residential communities in South Africa [18]. 

The effects of Mn on developmental stages in children have also been widely reported. A cross-sectional study in Brazil found negative associations between Mn hair levels and cognitive performance in children and caregivers as measured by Full-Scale IQ and Raven’s Progressive Matrix, respectively [19]. The Mn concentrations observed in this population were considered above average; nevertheless, a different study based in Canada found similar results with lower concentrations. Bouchard and co-workers found significant positive associations between Mn hair levels and hyperactive and oppositional behavior in children exposed to naturally high Mn levels in tap water [20]. Alternatively, Mn levels have been found to have a biphasic effect, where prenatal Mn is beneficial for adolescent cognition but has toxic effects at later time windows [21,22]. While these examples suggest an association between Mn exposure and cognitive function in adults and children, few data explore the effects of lower-level Mn on cognitive function in elderly individuals. 

This study investigated possible associations between Mn exposure and cognitive function using a representative U.S. elderly population obtained from the National Health and Examination Survey (NHANES). NHANES uses a complex sampling design and over samples persons 60 and older, as well as minority groups, to obtain an accurate representation of the United States population. Covariates such as race/ethnicity are important to consider as research suggests disadvantages in education and socioeconomic status across groups may influence cognitive function [23,24]. These same factors may increase one’s risk of exposure to other toxicants that may exacerbate the effects of Mn exposure. We used exposure biomarkers for Mn, including blood and urine, obtained from NHANES 2011–2012 and 2013–2014 cycles to conduct linear regression analyses, adjusting for several covariates to determine their relationship to cognitive function. 

## 2. Materials and Methods

### 2.1. Study Deseign and Data Sources

A cross-sectional study design was used to assess the effects of Mn exposure on cognitive function. The data were obtained from NHANES, a program for the National Center for Health Statistics (NCHS), a division of the Center for Disease Control and Prevention (CDC). NHANES is designed to assess the health and nutritional status of different populations across the United States by collecting data through a combination of health interviews and physical examinations. The health interviews include demographic, socioeconomic, and health-related questions, while the physical examinations include cognitive and physiological measurements performed by medical professionals. Two study samples for blood and urine were used of 2068 and 950 participants, respectively. Participants were selected using a complex, multistage probability design with appropriate weights to obtain a representative sample of the US population. Data from the 2011–2012 and 2013–2014 cycles were merged to conduct the analyses. The inclusion criteria consisted of individuals 60 years of age or older living in the US who had completed the Consortium to Establish a Registry for Alzheimer’s Disease (CERAD), Animal Fluency and Digital Symbol Substitution Test (DSST) test scores and also had measurements of blood and urine Mn levels.

### 2.2. Cognitive Function Assessments

Measurements of cognitive function were collected through at-home patient interviews using three different tests of cognitive performance: (1) word learning and recall modules from the CERAD, (2) the Animal Fluency test, and (3) the DSST. The CERAD assessed immediate and delayed learning ability for new verbal information [25]. It consisted of three learning trials and a delayed recall. Participants were instructed to read aloud 10 unrelated words and were immediately asked to recall as many words as possible. The order of the 10 words was changed in each trial. The delayed recall was assessed after the Animal Fluency and DSST tests were finished and about 8–10 min after the start of the CERAD’s learning trials. The final results included two scores—one for immediate word learning and another for delayed recall. The Animal Fluency test assessed categorical verbal fluency [26]. Participants were asked to name as many animals as possible in one minute, and a point was given for each animal named. The DSST assessed processing speed, sustained attention, and working memory [27]. Participants were asked to fill out a form that had a key with nine numbers paired with symbols. Participants were given two minutes to copy the corresponding symbols, and a point was given for each correct match. While higher test scores indicate better cognitive performance, these tests were not meant to replace a medical diagnosis but to examine the association of cognitive function with Mn exposure. 

### 2.3. Manganese Exposure Assessments

Single blood and urine samples used for analysis were collected at mobile examination centers (MECs) and shipped to the CDC’s Division of Laboratory Sciences in Atlanta, Georgia, for analysis. Once in the lab, manganese concentrations were determined using inductively coupled plasma mass spectrometry, and the resulting electrical signals were used to determine the concentration of the element [28]. The lower detection limit for blood Mn was 0.99 µg/L, and a fill value was used in the cases where the result was below the limit of detection; this value was the lower limit of detection divided by the square root of 2. The limit of detection for urinary Mn was 0.13 µg/L, and, likewise, a fill value was used in the cases where the result was below the limit of detection; this value was the lower limit of detection divided by the square root of 2. 

### 2.4. Covariates

Multiple covariates were included in the analysis to rule out any possible confounding factors or variables related to cognitive function or Mn exposure. These variables include age (60–64, 65–69, 70–74, 75–79, and 80 and older), gender (male and female), race/ethnicity (Hispanic, Non-Hispanic White, Non-Hispanic and Other Race), level of education (<high school, high school, >high school), poverty index ratio (PIR), marital status (Married/Living with a partner, Widowed/Divorced/Separated and Never Married), alcohol consumption (<12 drinks per year and >12 drinks per year), hypertension (yes and no), diabetes (yes and no), coronary heart disease (yes and no) and stroke (yes and no). 

### 2.5. Statistical Analysis

SAS/STATv14.2 was used for statistical analyses. NHANES data includes sampling weights incorporated into the analyses to adjust for possible biases such as unequal distribution poststratification and nonresponse. A new sampling weight was constructed for the merged 2011–2012 and 2013–2014 data cycles, following the publicly available protocol on the CDC’s website (CDC, 2021). The measurements of cognitive function are subject to floor and ceiling effects due to the wide range in function among the elderly population [1]. A composite cognitive z-score was created by averaging the standardized measurements of cognitive function to control for these effects, and the Kolmogorov–Smirnov test was used to assess normality. Stratified analyses between measurements of cognitive function were used to determine differences between tests. A descriptive analysis was performed to determine the distributions of demographic characteristics for individuals who completed all cognitive function tests. Univariate linear regression analyses were conducted to determine associations between the composite outcome z-score and individual covariates. Linear regression models were used to assess the relationship between Mn and the composite outcome z-score while adjusting for covariates. Any individuals with missing data were excluded from the respective models. Results were considered statistically significant at an alpha level of 0.05 and reported per 10 unit change in Mn level.

## 3. Results

Blood and urinary Mn data were analyzed as two separate cohorts to increase power. From the blood Mn cohort, 2068 participants had available data on cognitive function out of 3110 participants evaluated. These participants were on average 69.1 years of age. There were 1011 males and 1057 females in the cohort (46% vs. 54%, respectively). The composite outcome z-score was approximately normally distributed based on the Kolmogorov–Smirnov test (*p*-value = 0.1273), had a minimum and maximum of −2.5 and 2.5, respectively, and had a mean of 0.24. The average concentration of blood Mn levels was 9.4 µg/L per participant; about 22% of participants fell below the limit of detection. Table 1 depicts the demographic composition of the participants who had data on all four tests of cognitive function as well as a blood Mn measurement. Covariates including gender, age, race/ethnicity, education, PIR, marital status, alcohol consumption, hypertension, diabetes, coronary artery disease, and stroke were all significantly associated with the composite outcome z-score (Table S1). Univariate analyses suggested the need for their adjustment in the regression models.

Upon stratification of cognitive function by cognitive task, a 10 unit increase in blood Mn was associated with a 0.2-point decrease in CERAD (immediate) in model 2, adjusted for gender, age, race/ethnicity, PIR, and marital status (*β* = −0.2, 95% CI −0.3 to −0.01) (Table 2). This association was no longer significant in models 3 and 4 after adjusting for alcohol consumption and comorbidities (Table 2). A 10 unit increase in blood Mn was also associated with a 0.8-point decrease in AF scores in all models, after adjusting for all covariates (*β* = −0.8, 95% CI −1.4 to −0.1) (Table 3). Blood Mn was not significantly associated with CERAD (delayed) or DSST scores (Tables S2 and S3). Blood Mn was not significantly associated with composite outcome z-score in unadjusted or adjusted linear regression models (Table S4).

From the urinary Mn cohort, 950 participants had available data on cognitive function out of 3110 participants evaluated. These participants were on average 69.3 years of age. There were 464 males and 486 females in the cohort (46% vs. 54%, respectively). The average concentration of urinary Mn levels was 0.19 µg/L per participant; about 64% of participants fell below the limit of detection. The high percentage of measurements in the urine cohort explains why the average concentration was so close to the limit of detection. This may further be explained by the fact that more than 90% of Mn is excreted into feces [29,30]. Table 4 depicts the demographic composition of the participants who had data on all four tests of cognitive function as well as a blood Mn measurement. Similar to the blood Mn cohort, all covariates in the urinary Mn cohort were significantly associated with the composite z-score and, therefore, adjusted for in the models (Table S5). 

Upon stratification of cognitive task, a 10 unit increase in urinary Mn was associated with a 2.4-point decrease in CERAD (immediate) after adjusting for all covariates in model 4 (*β* = −2.4, 95% CI −4.1 to −0.7) (Table 5). A 10 unit increase in urinary Mn was also associated with a 30-point decrease in DSST scores in models 1 to 3 (*β* = −30, 95% CI −40 to −10); however, significance diminished when adjusting for all covariates in model 4 (Table 6). Urinary Mn was not significantly associated with CERAD (delayed) or AF scores (Tables S6 and S7). Urinary Mn was also not significantly associated with composite outcome z-score in unadjusted or adjusted linear regression models (Table S8).

**Figure 1:**
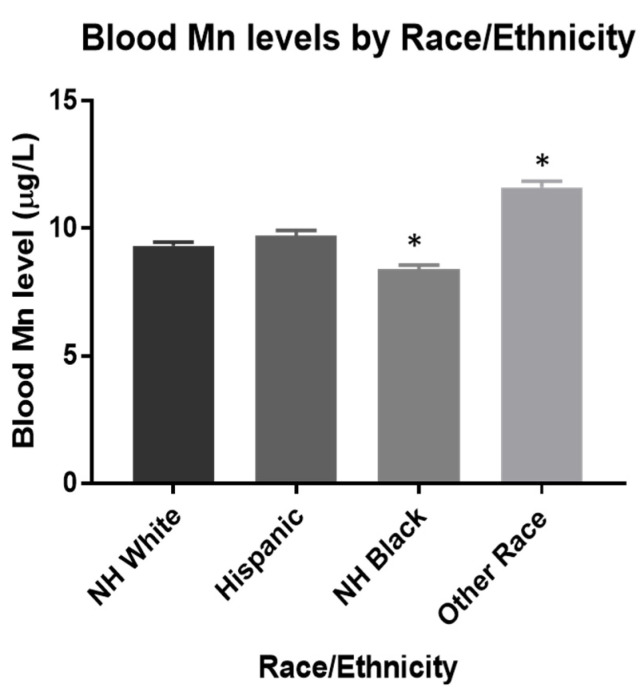
Estimated mean blood Mn levels were 9.3, 9.7, 8.4, and 11.6 μg/L for NH-White (*n* = 988), Hispanic (*n* = 385), NH-Black (*n* = 491), and Other Race (*n* = 204), respectively. * *p* < 0.0001. Data were analyzed by One-way ANOVA with Tukey’s *post-hoc* test holding NH White as the control. The limit of detection used was 0.99 μg/L.

**Figure 2:**
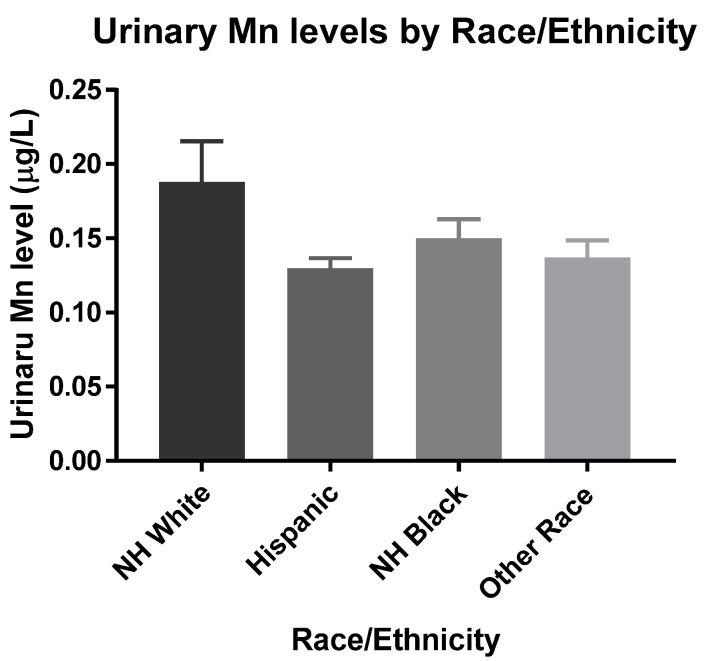
Estimated mean urinary Mn levels were 0.19, 0.13, 0.15, and 0.14 μg/L for NH-White (*n* = 451), Hispanic (*n* = 176), NH-Black (*n* = 225), and Other Race (*n* = 98), respectively. Data were analyzed by One-way ANOVA with Tukey’s *post-hoc* test holding NH White as the control. The limit of detection used was 0.13 μg/L.

Univariate analyses between all covariates and z-score showed that race/ethnicity had the most substantial effect on cognitive function (Tables S1 and S5). To further investigate this, the mean blood and urinary Mn levels were compared across each race/ethnicity. Figure 1 shows Other Races had significantly higher blood Mn levels when compared to NH-Whites, while it was significantly lower in NH-Blacks. Hispanics did not have significant differences in mean blood Mn levels compared to NH-Whites (Figure 1). These racial and ethnic differences are similar to those previously found and may be explained by dietary differences [2]. Consumption of tea, which has a considerable amount of Mn, is believed to play a significant role in Mn intake [31]. Additionally, there were no significant differences observed in mean urinary Mn levels across all four groups (Figure 2). 

Lastly, linear regression analysis showed no significant associations between blood Mn levels and urinary Mn levels in unadjusted and adjusted models (Table S9). 

## 4. Discussion

To our knowledge, this is the first study to investigate possible associations between blood and urinary Mn levels and cognitive function in an elderly US-based population. We found significant inverse associations between blood and urinary Mn levels with cognitive function when stratified by four cognitive function tests, while there was no significant association between blood and urinary Mn levels and composite outcome z-score. Specifically, blood Mn was inversely associated with CERAD (immediate) after adjusting for gender, age, race/ethnicity, PIR, and marital status; this association is no longer significant after adjusting for medical history. In addition, blood Mn was inversely associated with AF scores after adjusting for all covariates. Finally, urinary Mn was inversely associated with CERAD (immediate) after adjusting for all covariates and inversely associated with DSST scores in models 1 to 3, while no longer significant after adjusting for medical history. Each cognitive function test measures different cognitive domains that are influenced by various brain regions and interactions among these regions, and these results may suggest a region-specific effect of Mn [25,26,27].

A similar NHANES study has previously reported an inverse association between urinary Mn levels and high blood pressure in adults [32]. Oulhote and co-workers reported similar differences in blood Mn levels across different race/ethnicities, indicating the importance of considering race/ethnicity as a confounding factor when conducting future studies [2]. Studies in children have reported high Mn exposure associated with poor childhood development characterized by motor skills and behavioral performance [33]. Furthermore, elevated Mn levels in private wells are associated with increased congenital disabilities [34]. Together, these data suggest that Mn may be implicated in a broad range of adverse effects that may influence cognitive function and highlight the need for further research on the mechanisms through which it induces toxic effects. 

The exact molecular mechanisms of Mn-induced neurotoxicity have not been fully established. It is believed that Mn exposure has a biphasic relationship with health outcomes, suggesting Mn serves as an essential nutrient at an optimal concentration but has toxic effects at low and high levels [35]. Mn is transported across the blood-brain barrier (BBB) via several pathways, such as the divalent metal transporter 1 (DMT1) and the transferrin receptor system and accumulates in Fe-rich regions of the basal ganglia [11,35]. Mutations in the Mn efflux and influx transporter genes SLC30A10 and SLC30A8 can alter Mn levels in the Golgi apparatus and induce neurotoxicity via aberrant vesicular trafficking [35,36]. Evidence suggests Mn may increase autophagy at higher levels, implicating a role in the degradation of protein aggregates associated with neurodegenerative diseases such as PD or Alzheimer’s disease AD [37]. Its implication in neurodegenerative diseases is further evidenced by its ability to alter mitochondrial function and induce dopaminergic cell loss [35]. Furthermore, Mn accumulation affects the metabolism of other metals, and Mn toxicity may indirectly lead to toxic increases of other metals as well [1]. Epidemiological studies investigating possible environmental associations between Mn and adverse effects can help provide further insight into prevention methods. 

A major strength of this study is the utility of a representative U.S.-based elderly population. NHANES is designed to oversample minority populations and apply adjusted weights to ensure representation. Nevertheless, the data reported in this study should be interpreted with caution based on several limitations. First, there were data excluded from both cohorts due to missing values. The final cohorts represent 66% and 31% of the participants considered for blood and urine analyses, respectively. Second, blood and urine Mn levels are generally thought to reflect acute exposure to Mn. The half-life of urinary Mn is believed to be about 30 h, while it is only about 2 h in the blood [38,39]. This difference may explain why there was no significant association found between blood Mn and urinary Mn levels as the renal route of excretion is only responsible for up to about 5% of Mn excretion [38,40]. The biologic half-life of Mn in the brain has been previously reported to be between 51–74 days, also suggesting that blood and serum levels may not be reflective of the total concentration of Mn in the body [41]. The average blood and urine Mn levels seen in our sample population also fall below the average Mn levels reported in the Agency for Toxic Substances and Disease Registry of 4–15 μg/L in blood and 1–8 μg/L in urine [42]. Moreover, previous studies have found urine to be a poor biomarker for Mn exposure [43]. Ellingsen and co-workers found weak associations between urinary Mn and inhalable aerosol fractions in a group of workers in Mn-alloy producing plants [44]. Future research should focus on using biomarkers that accurately depict long-term exposure. For example, Laohaudomchok and co-workers found toenails to be a measure of Mn levels up to 12 months after exposure [45]. Ideally, an integrated approach that considers multiple biomarkers for Mn exposure provides a more accurate representation of the total body burden of exposure [46]. 

Lastly, only biomarkers of Mn were used, and it is possible that exposure to other neurotoxic agents may have confounded the results. For example, other metals such as Al, Se, Pb, Zn, Cd, and Hg have all been implicated in a broad range of neurodegenerative disorders and may share similar routes of exposure to Mn [47,48,49]. Zn transporters, ZIP8 and ZIP14, have been suggested to also be involved in Mn transport pathways, indicating that co-exposure of these two metals may have combinatorial effects [50]. Cognitive dysfunction is a common clinical presentation of many neurodegenerative diseases. These diseases are multifaceted and co-exposure of a variety of environmental factors such as pesticides or particles found in air pollution may further affect the role of Mn in cognitive function. 

## 5. Conclusions

Collectively, these findings suggest that increased blood and urinary Mn levels are associated with poorer cognitive function in an elderly US population. These data further support the need for preventative approaches to reduce excess Mn exposure. Future studies incorporating biomarker matrices that are more stable and using cohorts with repeated samples available will be important in determining the impact of chronic, lower-level Mn exposure on cognitive function.

## Figures and Tables

**Table 1:** Characteristics of the Mn-Blood study population (*n* = 2068).

Variables	N	Weighted Sample	(%) *	Variables	N	Weighted Sample	Mean (SE)	Median (IQR)
Gender								
Male	1011	1,7508,127	46	Age (year)	2068	38,452,145	69.1 (0.24)	67.7 (11)
Female	1057	20,944,017	54	Blood Mn (µg/L)	2068	38,452,145	9.4 (0.08)	8.8 (3.8)
Race/Ethnicity				CERAD learning	2068	38,452,145	6.5 (0.1)	6.5 (2)
Hispanic	385	2,618,094	7	CERAD recall	2068	38,452,145	6.1 (0.1)	5.8 (3.2)
NH White	988	30,935,957	80	Animal Fluency	2068	38,452,145	18 (0.2)	17 (7.5)
NH Black	491	2,980,219	8	DSST	2068	38,452,145	52 (0.7)	53 (24)
Other Race	204	1,917,874	5	z-score	2068	38,452,145	0.24 (0.04)	0.28 (1.1)
Education								
<High School	531	6,133,440	16					
High School	490	8,783,404	23					
>High School	1046	23,530,632	61					
Missing	1							
PIR								
≤0.99	333	3,298,332	9					
≥1	1550	32,672,780	91					
Missing	185							
Marital Status								
Married/Living with partner	1201	25,353,317	69					
Widowed/Divorced/ Separated	742	11,403,761	31					
Missing	125							
Alcohol Consumption								
>12 drinks/year	1397	27,847,784	73					
<12 drinks/year	640	10,098,855	27					
Missing	31							
HTN								
Yes	1262	21,869,127	57					
No	804	16,514,849	43					
Missing	2							
DM								
Yes	472	7,293,920	20					
No	1508	29,747,613	80					
Missing	88							
CAD								
Yes	172	3,086,811	8					
No	1886	35,263,550	92					
Missing	10							
Stroke								
Yes	138	2,341,705	6					
No	1926	36,057,144	94					
Missing	4							

* Weighted percentage, mean, SE, median, and IQR. CERAD, Consortium to Establish a Registry for Alzheimer’s Disease; DSST, Digital Symbol Substitution Test; HTN, hypertension; DM, diabetes mellitus; CAD, coronary artery disease.

**Table 2:** Mn-Blood levels (continuous) in relation to CERAD (immediate).

	Model 1 (*n* = 2068) *		Model 2 (*n* = 1772) †		Model 3 (*n* = 1744) ‡		Model 4 (*n* = 1650) §	
Variables	*β* (95% CI) ^¶^	*p*-Value	*β* (95% CI) ^¶^	*p*-Value	*β* (95% CI) ^¶^	*p*-Value	*β* (95% CI) ^¶^	*p*-Value
Mn	−0.03 (−0.2 to 0.2)	0.714	−0.2 (−0.3 to −0.01)	0.04	−0.2 (−0.3 to 0.01)	0.07	−0.2 (−0.3 to 0.03)	0.09
Gender								
Male			referent	referent	referent	referent	referent	referent
Female			6 (4 to 7)	<0.0001	6 (4 to 8)	<0.0001	6 (4 to 8)	<0.0001
Age (year)			−0.8 (−0.9 to −0.7)	<0.0001	−0.8 (−0.9 to −0.6)	<0.0001	−0.8 (−0.9 to −0.6)	<0.0001
Race/Ethnicity								
NH White			referent	referent	referent	referent	referent	referent
Hispanic			−5 (−8 to −2)	0.005	−4 (−8 to −1)	0.01	−4 (−8 to −1)	0.01
NH Black			−2 (−4 to 1)	0.18	−1 (−4 to 1)	0.31	−0.5 (−3 to 2)	0.65
Other Race			−2 (−5 to 1)	0.21	−2 (−5 to 1)	0.23	−1 (−4 to 2)	0.32
Education								
>High School			referent	referent	referent	referent	referent	referent
High School			−4 (−6 to −2)	0.0004	−4 (−6 to −2)	0.001	−4 (−6 to −2)	0.001
<High School			−6 (−10 to −2)	0.004	−6 (−10 to −2)	0.01	−5 (−10 to −1)	0.01
PIR								
≤0.99			referent	referent	referent	referent	referent	referent
≥1			5 (3 to 7)	<0.0001	5 (3 to 7)	<0.0001	5 (3 to 7)	<0.0001
Marital Status								
Married/Living with partner			referent	referent	referent	referent	referent	referent
Widowed/Divorced/ Separated			−1 (−4 to 1)	0.31	−1 (−4 to 1)	0.34	0.8 (−3 to 2)	0.48
Alcohol Consumption								
<12 drinks/year					referent	referent	referent	referent
>12 drinks/year					1 (−1 to 4)	0.17	1 (−1 to 4)	0.19
HTN								
No							referent	referent
Yes							−1 (−3 to 1)	0.33
DM								
No							referent	referent
Yes							−2 (−4 to −1)	0.002
Stroke								
No							referent	referent
Yes							−0.1 (−3 to 3)	0.97
CAD								
No							referent	referent
Yes							−2 (−6 to 3)	0.43

* Unadjusted. † Adjusted for age, gender, ethnicity, education, PIR, and marital status. ‡ Adjusted for age, gender, ethnicity, education, PIR, marital status, and alcohol consumption. § Adjusted for age, gender, ethnicity, education, PIR, marital status, alcohol consumption, HTN, DM, stroke, and CAD. ^¶^ Weighted *β* and 95% Confidence Interval.

**Table 3:** Mn-Blood levels (continuous) in relation to AF.

	Model 1 (*n* = 2068) *		Model 2 (*n* = 1772) †		Model 3 (*n* = 1744) ‡		Model 4 (*n* = 1650) §	
Variables	*β* (95% CI) ^¶^	*p*-Value	*β* (95% CI) ^¶^	*p*-Value	*β* (95% CI) ^¶^	*p*-Value	*β* (95% CI) ^¶^	*p*-Value
Mn	0.4 ( −1 to 0.2)	0.21	−0.8 (−1 to −0.1)	0.02	−0.7 (−1 to −0.1)	0.02	−0.8 (−1 to −0.1)	0.03
Gender								
Male			referent	referent	referent	referent	referent	referent
Female			−0.5 (−9 to 8)	0.91	0.9 (−8 to 10)	0.83	2 (−7 to 10)	0.64
Age (year)			−3 (−3 to −2)	<0.0001	−3 (−3 to −2)	<0.0001	−2 (−3 to −2)	<0.0001
Race/Ethnicity								
NH White			referent	referent	referent	referent	referent	referent
Hispanic			−23 (−30 to −20)	<0.0001	−23 (−30 to −20)	<0.0001	−23 (−30 to −20)	<0.0001
NH Black			−33 (−40 to −30)	<0.0001	−32 (−40 to −20)	<0.0001	−31 (−40 to −20)	<0.0001
Other Race			−24 (−30 to −10)	0.0005	−23 (−40 to −10)	0.0007	−21 (−30 to −10)	0.003
Education								
>High School			referent	referent	referent	referent	referent	referent
High School			−29 (−40 to −20)	<0.0001	−28 (−40 to −20)	<0.0001	−28 (−40 to −20)	<0.0001
<High School			−33 (−40 to −30)	<0.0001	−32 (−40 to −30)	<0.0001	−31 (−40 to −20)	<0.0001
PIR								
≤0.99			referent	referent	referent	referent	referent	referent
≥1			12 (2 to 20)	0.02	11 (2 to 20)	0.02	11 (1 to 20)	0.03
Marital Status								
Married/Living with partner			referent	referent	referent	referent	referent	referent
Widowed/Divorced/Separated			−0.4 (−9 to 8)	0.93	−0.7 (−10 to 8)	0.88	−1 (−10 to 8)	0.82
Alcohol Consumption								
<12 drinks/year					referent	referent	referent	referent
>12 drinks/year					7 (0.8 to 10)	0.03	7 (0.8 to 10)	0.03
HTN								
No							referent	referent
Yes							−6 (−10 to 0.2)	0.06
DM								
No							referent	referent
Yes							−8 (−10 to −1)	0.03
Stroke								
No							referent	referent
Yes							−6 (−20 to 5)	0.27
CAD								
No							referent	referent
Yes							−9 (−20 to 4)	0.16

* Unadjusted. † Adjusted for age, gender, ethnicity, education, PIR, and marital status. ‡ Adjusted for age, gender, ethnicity, education, PIR, marital status, and alcohol consumption. § Adjusted for age, gender, ethnicity, education, PIR, marital status, alcohol consumption, HTN, DM, stroke, and CAD. ^¶^ Weighted *β* and 95% Confidence Interval.

**Table 4:** Characteristics of the Mn-Urine study population (*n* = 950).

Variables	N	Weighted Sample	(%) *	Variables	N	Weighted Sample	Mean (SE)	Median (IQR)
Gender								
Male	464	7,702,844	46	Age (year)	950	16,795,626	69.3 (0.3)	67.9 (10.7)
Female	486	9,092,783	54	Urinary Mn (µg/L)	950	16,795,626	0.19 (0.03)	0.09 (0.07)
Race/Ethnicity				CERAD learning	950	16,795,626	6.6 (0.01)	6.6 (2.1)
Hispanic	176	1,216,571	7	CERAD recall	950	16,795,626	6.3 (0.1)	6.1 (3.3)
NH White	451	13,351,140	79	Animal Fluency	950	16,795,626	18 (0.3)	16 (8)
NH Black	225	1,438,019	9	DSST	950	16,795,626	52 (0.8)	51 (25)
Other Race	98	789,895	5	z-score	950	16,795,626	0.23 (0.04)	0.29 (1.1)
Education								
<High School	259	2,847,794	17					
High School	209	3,422,549	20					
>High School	480	10,513,695	63					
Missing	2							
PIR								
≤0.99	166	1,539,528	10					
≥1	693	13,934,209	90					
Missing	91							
Marital Status								
Married/Living with partner	557	10,964,908	65					
Widowed/Divorced/Separated	336	5,008,167	30					
Never married	55	810,401	5					
Missing	2							
Alcohol Consumption								
>12 drinks/year	644	12,105,591	73					
<12 drinks/year	288	4,409,341	27					
Missing	18							
HTN								
Yes	589	96,29,710	57					
No	361	7,165,916	43					
DM								
Yes	214	3,081,562	19					
No	694	12,955,019	81					
Missing	42							
CAD								
Yes	76	1,380,980	8					
No	869	15,348,084	92					
Missing	5							
Stroke								
Yes	49	760,890	5					
No	900	16,002,161	95					
Missing	1							

* Weighted percentage, mean, SE, median, and IQR. CERAD, Consortium to Establish a Registry for Alzheimer’s Disease; DSST, Digital Symbol Substitution Test; HTN, hypertension; DM, diabetes mellitus; CAD, coronary artery disease.

**Table 5:** Mn-Urine levels (continuous) in relation to CERAD (immediate).

	Model 1 (*n* = 2068) *		Model 2 (*n* = 1772) †		Model 3 (*n* = 1744) ‡		Model 4 (*n* = 1650) §	
Variables	*β* (95% CI) ^¶^	*p*-Value	*β* (95% CI) ^¶^	*p*-Value	*β* (95% CI) ^¶^	*p*-Value	*β* (95% CI) ^¶^	*p*-Value
Mn	−0.7 (−2 to 0.2)	0.16	−1 (−2 to 0.3)	0.12	−1 (−2 to 0.2)	0.09	−2 (−4 to −0.7)	0.01
Gender								
Male			referent	referent	referent	referent	referent	referent
Female			7 (5 to 9)	<0.0001	7 (5 to 9)	<0.0001	7 (5 to 9)	<0.0001
Age (year)			−0.8 (−1 to −0.6)	<0.0001	−0.8 (−1 to −0.5)	<0.0001	−0.7 (−1 to −0.5)	<0.0001
Race/Ethnicity								
NH White			referent	referent	referent	referent	referent	referent
Hispanic			−7 (−10 to −4)	<0.0001	−7 (−10 to −4)	<0.0001	−6 (−10 to −4)	0.0001
NH Black			0.2 (−2 to 3)	0.89	0.5 (−2 to 3)	0.67	0.7 (−2 to 4)	0.6
Other Race			−5 (−10 to 1)	0.1	−4 (−10 to 11)	0.12	−4 (−10 to 1)	0.13
Education								
>High School			referent	referent	referent	referent	referent	referent
High School			−4 (−10 to −4)	0.0003	−4 (−6 to −2)	0.001	−3 (−6 to −1)	0.003
<High School			−7 (−10 to −4)	0.0002	−7 (−10 to 3)	0.0004	−6 (−10 to −3)	0.001
PIR								
≤0.99			referent	referent	referent	referent	referent	referent
≥1			1 (−2 to 5)	0.4	1 (−2 to −5)	0.51	0.9 (−3 to 5)	0.59
Marital Status								
Married/Living with partner			referent	referent	referent	referent	referent	referent
Widowed/Divorced/Separated			−2 (−5 to 0.6)	0.12	−2 (−5 to 0.5)	0.11	−2 (−5 to 0.6)	0.13
Alcohol Consumption								
<12 drinks/year					referent	referent	referent	referent
>12 drinks/year					2 (−1 to 5)	0.19	2 (−1 to 5)	0.24
HTN								
No							referent	referent
Yes							−2 (−5 to 0.5)	0.1
DM								
No							referent	referent
Yes							−1 (−4 to 1)	0.27
Stroke								
No							referent	referent
Yes							−3 (−7 to 2)	0.27
CAD								
No							referent	referent
Yes							0.3 (−6 to 7)	0.93

* Unadjusted. † Adjusted for age, gender, ethnicity, education, PIR, and marital status. ‡ Adjusted for age, gender, ethnicity, education, PIR, marital status, and alcohol consumption. § Adjusted for age, gender, ethnicity, education, PIR, marital status, alcohol consumption, HTN, DM, stroke, and CAD. ^¶^ Weighted *β* and 95% Confidence Interval.

**Table 6:** Mn-Urine levels (continuous) in relation to DSST.

	Model 1 (*n* = 2068) *		Model 2 (*n* = 1772) †		Model 3 (*n* = 1744) ‡		Model 4 (*n* = 1650) §	
Variables	*β* (95% CI) ^¶^	*p*-Value	*β* (95% CI) ^¶^	*p*-Value	*β* (95% CI) ^¶^	*p*-Value	*β* (95% CI) ^¶^	*p*-Value
Mn	−20 (−40 to −10)	0.003	−30 (−40 to −10)	0.002	−30 (−40 to −10)	0.0002	−30 (−70 to 10)	0.13
Gender								
Male			referent	referent	referent	referent	referent	referent
Female			70 (40 to 100)	<0.0001	80 (50 to 100)	<0.0001	70 (50 to 100)	<0.0001
Age (year)			−10 (−12 to −9)	<0.0001	−10 (−12 to −9)	<0.0001	−10 (−2 to −9)	<0.0001
Race/Ethnicity								
NH White			referent	referent	referent	referent	referent	referent
Hispanic			−120 (−150 to −90)	<0.0001	−120 (−150 to −90)	<0.0001	−120 (−150 to −90)	<0.0001
NH Black			−110 (−130 to −80)	<0.0001	−100 (−120 to −80)	<0.0001	−100 (−130 to −70)	<0.0001
Other Race			−20 (−60 to 20)	0.29	−10 (−50 to 20)	0.45	−20 (−60 to 20)	0.38
Education								
>High School			referent	referent	referent	referent	referent	referent
High School			−80 (−110 to −40)	<0.0001	−70 (−110 to −40)	0.0003	−60 (−100 to −80)	0.002
<High School			−130 (−160 to −100)	<0.0001	−120 (−150 to −90)	<0.0001	−110 (−140 to −80)	<0.0001
PIR								
≤0.99			referent	referent	referent	referent	referent	referent
≥1			80 (40 to 110)	<0.0001	70 (40 to 110)	0.0002	70 (40 to 110)	0.0002
Marital Status								
Married/Living with partner			referent	referent	referent	referent	referent	referent
Widowed/Divorced/Separated			−20 (−50 to 10)	0.09	−30 (−50 to 10)	0.06	−30 (−60 to 10)	0.04
Alcohol Consumption								
<12 drinks/year					referent	referent	referent	referent
>12 drinks/year					40 (10 to 70)	0.01	30 (10 to 60)	0.03
HTN								
No							referent	referent
Yes							−2 (−30 to 20)	0.88
DM								
No							referent	referent
Yes							−60 (−100 to −30)	0.0003
Stroke								
No							referent	referent
Yes							−20 (−70 to 40)	0.5
CAD								
No							referent	referent
Yes							−50 (−100 to 10)	0.1

* Unadjusted. † Adjusted for age, gender, ethnicity, education, PIR, and marital status. ‡ Adjusted for age, gender, ethnicity, education, PIR, marital status, and alcohol consumption. § Adjusted for age, gender, ethnicity, education, PIR, marital status, alcohol consumption, HTN, DM, stroke, and CAD. ^¶^ Weighted *β* and 95% Confidence Interval.

## Data Availability

The NHANES data is publicly available at https://www.cdc.gov/nchs/nhanes/genetics/genetic_participants.htm, accessed on 3 March 2022.

## Supplementary Materials

The following supporting information can be downloaded at: https://www.mdpi.com/article/10.3390/toxics10040191/s1, Table S1: Mn-Blood univariate analyses of the association between z-score and covariates (*n* = 2068), Table S2: Mn-Blood levels (continuous) in relation to CERAD (delayed), Table S3: Mn-Blood levels (continuous) in relation to DSST, Table S4: Mn-Blood levels (continuous) in relation to z-score, Table S5: Mn-Urine univariate analyses of the association between z-score and covariates (*n* = 950), Table S6: Mn-Urine levels (continuous) in relation to CERAD (delayed), Table S7: Mn-Urine levels (continuous) in relation to AF, Table S8: Mn-Urine levels (continuous) in relation to cognitive function, Table S9: Mn-Blood levels (continuous) in relation to Mn-Urine.
