# Supplementary material for: Relationship of Blood and Urinary Manganese Levels with Cognitive Function in Elderly Individuals in the United States by Race/Ethnicity, NHANES 2011–2014
Source: Toxics. 2022 Apr 14;10(4):191. doi: 10.3390/toxics10040191 (PMC9025725; doi:10.3390/toxics10040191)
Supplement: Supplementary file 1 [file toxics-10-00191-s001.zip › toxics-1646150-supplementary-done.pdf]

toxics

Supplementary Materials: Relationship of Blood and Urinary Manganese Levels with Cognitive Function in Elderly Individuals in the United States by Race/Ethnicity, NHANES 2011–2014

Arturo J. Barahona, Zoran Bursac, Emir Veledar, Roberto Lucchini, Kim Tieu and Jason R. Richardson

Table S1. Mn-Blood univariate analyses of the association between z-score and covariates (n = 2068).

Variables	Score Mean (SE)*	F value	P-value
Gender		14.85	0.0005
Male	0.16 (0.04)		
Female	0.32 (0.04)		
Age (year)	-	−20.38**	<0.0001
Race/ethnicity		25.1	<0.0001
Hispanic	−0.28 (0.05)		
NH White	0.33 (0.05)		
NH Black	−0.13 (0.05)		
Other race	0.14 (0.06)		
Education		79.45	<0.0001
<High School	−0.34 (0.05)		
High School	0.05 (0.05)		
>High School	0.47 (0.04)		
PIR		68.62	<0.0001
<=0.99	−0.30 (0.06)		
>=1	0.31 (0.04)		
Marital Status		14.18	0.0007
Married/Living with partner	0.31 (0.05)		
Widowed/Divorced/Separated	0.10 (0.04)		
Alcohol Consumption		27.46	<0.0001
>12 drinks/yr	0.32 (0.05)		
<12 drinks/yr	0.04 (0.03)		
HTN		25.78	<0.0001
Yes	0.15 (0.04)		
No	0.38 (0.04)		
DM		26.97	<0.0001
Yes	−0.03 (0.04)		
No	0.31 (0.05)		
CAD		28.41	<0.0001
Yes	−0.11 (0.06)		
No	0.28 (0.04)		
Stroke		13.24	0.001
Yes	−0.16 (0.10)		
No	0.27 (0.04)		

*Weighted mean and SE; **t-score.

Table S2. Mn-Blood levels (continuous) in relation to CERAD (delayed).

	Model 1 (*n* = 2068)*		Model 2 (*n* = 1772)†		Model 3 (*n* = 1744)‡		Model 4 (*n* = 1650)§	
Variables	*β* (95% CI)¶	*p*-value	*β* (95% CI)¶	*p*-value	*β* (95% CI)¶	*p*-value	*β* (95% CI)¶	*p*-value
Mn	0.2 (−0.1 to 0.4)	0.2	−0.03 (−0.3 to 0.2)	0.77	−0.04 (−0.3 to 0.2)	0.76	−0.04 (−0.3 to 0.2)	0.74
Gender								
Male			referent	referent	referent	referent	referent	referent
Female			8 (6 to 10)	<0.0001	8 (6 to 10)	<0.0001	0.8 (0.5 to 1)	<0.0001
Age (year)			−1 (−14 to −10)	<0.0001	−12 (−14 to −1)	<0.0001	−1 (−14 to −0.9)	<0.0001
Race/Ethnicity								
NH White			referent	referent	referent	referent	referent	referent
Hispanic			−7 (−10 to −2)	0	−7 (−10 to −2)	0.01	−7 (−10 to −2)	0.01
NH Black			−4 (−7 to −0.4)	0.03	−3 (−7 to −0.1)	0.05	−3 (−6 to 1)	0.15
Other Race			1.3 (−5 to 8)	0.7	1 (−6 to 8)	0.7	1 (−5 to 8)	0.63
Education								
>High School			referent	referent	referent	referent	referent	referent
High School			−5 (−9 to −1)	0.01	−5 (−9 to −1)	0.01	−6 (−10 to −2)	0.005
<High School			−5 (−10 to −0.01)	0.05	−5 (−10 to −0.1)	0.05	−5 (−10 to 01)	0.05
PIR								
<=0.99			referent	referent	referent	referent	referent	referent
>=1			5 (1 to 8)	0.01	5 (1 to 8)	0.02	4 (1 to 8)	0.02
Marital Status								
Married/Living with partner			referent	referent	referent	referent	referent	referent
Widowed/Divorced/ Separated			−0.9 (−5 to 3)	0.6	−1 (−5 to 3)	0.57	−0.9 (−4 to 3)	0.6
Alcohol Consumption								
<12 drinks/yr					referent	referent	referent	referent
>12 drinks/yr					0.7 (−2 to 3)	0.6	0.2 (−2 to 3)	0.87
HTN								
No							referent	referent
Yes							−0.7 (−4 to 2)	0.63
DM								
No							referent	referent

Yes	−4 (−7 to −2)	0.002
Stroke		
No	referent	referent
Yes	−1 (−7 to 5)	0.71
CAD		
No	referent	referent
Yes	−4 (−10 to 2)	0.2

*Unadjusted.† Adjusted for age, gender, ethnicity, education, PIR, and marital status. ‡ Adjusted for age, gender, ethnicity, education, PIR, marital status, and alcohol consumption. § Adjusted for age, gender, ethnicity, education, PIR, marital status, alcohol consumption, HTN, DM, stroke, and CAD. ¶ Weighted β and 95% Confidence Interval.

Table S3. Mn-Blood levels (continuous) in relation to DSST.

	Model 1 (*n* = 2068)*		Model 2 (*n* = 1772)†		Model 3 (*n* = 1744)‡		Model 4 (*n* = 1650)§	
Variables	*β* (95% CI)¶	*P*-value	*β* (95% CI)¶	*P*-value	*β* (95% CI)¶	*P*-value	*β* (95% CI)¶	*P*-value
Mn	0.5 (−2 to 3)	0.69	−1 (−4 to 1)	0.26	−1 (−4 to 1)	0.37	−1 (−4 to 1)	0.4
Gender								
Male			referent	referent	referent	referent	referent	referent
Female			50 (40 to 60)	<0.0001	60 (40 to 70)	<0.0001	50 (40 to 70)	<0.0001
Age (year)			−10 (−10 to 10)	<0.0001	−10 (−10 to −8)	<0.0001	−10 (−11 to −8)	<0.0001
Race/Ethnicity								
NH White			referent	referent	referent	referent	referent	referent
Hispanic			−120 (−140 to −100)	<0.0001	−120 (−140 to −100)	<0.0001	−120 (−150 to −100)	<0.0001
NH Black			−120 (−140 to −100)	<0.0001	−120 (−1300 to −10)	<0.0001	−110 (−130 to −90)	<0.0001
Other Race			−30 (−60 to −10)	0.01	−30 (−60 to −10)	0.02	20 (−50 to 10)	0.03
Education								
>High School			referent	referent	referent	referent	referent	referent
High School			−80 (−100 to −60)	<0.0001	−80 (−90 to −60)	<0.0001	−70 (−100 to −50)	<0.0001
<High School			−140 (−160 to −110)	<0.0001	−130 (−150 to −110)	<0.0001	−130 (−15 to −10)	<0.0001
PIR								
<=0.99			referent	referent	referent	referent	referent	referent
>=1			80 (50 to 110)	<0.0001	8 0(40 to 110)	<0.0001	80 (50 to 110)	<0.0001
Marital Status								
Married/Living with partner			referent	referent	referent	referent	referent	referent

Widowed/Divorced/ Separated	−10 (−30 to 5)	0.17	−10 (−30 to 10)	0.15	−10 (−30 to 10)	0.24
Alcohol Consumption						
<12 drinks/yr			referent	referent	referent	referent
>12 drinks/yr			30 (10 to 40)	0.004	20 (10 to 40)	0.01
HTN						
No					referent	referent
Yes					−10 (−30 to 10)	0.09
DM						
No					referent	referent
Yes					−40 (−60 to −20)	0.001
Stroke						
No					referent	referent
Yes					−10 (−40 to 20)	0.37
CAD						
No					referent	referent
Yes					−40 (−70 to −20)	0.004

*Unadjusted.† Adjusted for age, gender, ethnicity, education, PIR, and marital status. ‡ Adjusted for age, gender, ethnicity, education, PIR, marital status, and alcohol consumption. § Adjusted for age, gender, ethnicity, education, PIR, marital status, alcohol consumption, HTN, DM, stroke, and CAD. ¶ Weighted β and 95% Confidence Interval.

Table S4. Mn-Blood levels (continuous) in relation to z-score.

	Model 1 (n = 2068)*		Model 2 (n = 1772)†		Model 3 (n = 1744)‡		Model 4 (n = 1650)§	
Variables	β (95% CI)¶	P-value	β (95% CI)¶	P-value	β (95% CI)¶	P-value	β (95% CI)¶	P-value
Mn	0.001 (−0.09 to 0.1)	0.9821	−0.1 (−0.1 to 0.01)	0.057	0.1 (−0.1 to 0.01)	0.081	−0.1 (−0.2 to 0.02)	0.101
Gender								
Male			referent	referent	referent	referent	referent	referent
Female			2.4 (2 to 3)	<0.0001	2.6 (2 to 4)	<0.0001	2.6 (2 to 3)	<0.0001
Age (year)			−0.5 (−1 to −0.5)	<0.0001	−0.5 (−0.5 to −0.4)	<0.0001	−0.5 (−0.6 to −0.4)	<0.0001
Race/Ethnicity								
NH White			referent	referent	referent	referent	referent	referent
Hispanic			−4.3 (−6 to −3)	<0.0001	−4.2 (−5 to −3)	<0.0001	−4.3 (−6 to −3)	<0.0001
NH Black			−3.9 (−5 to −3)	<0.0001	−3.7 (−0.5 to −3)	<0.0001	−3.4 (−0.4 to −2)	<0.0001
Other Race			−1.8 (−3 to −0.6)	0.006	−1.7 (−3 to −0.4)	0.01	−1 (−3 to −0.3)	0.019

Education						
>High School	referent	referent	referent	referent	referent	referent
High School	−3.6 (−5 to −3)	<0.0001	−3 (−0.5 to −0.3)	<0.0001	−3.5 (−4 to −3)	<0.0001
<High School	−5 (−6 to −4)	<0.0001	−4.9 (−6 to −3)	<0.0001	−4.7 (−6 to −3)	<0.0001
PIR						
<=0.99	referent	referent	referent	referent	referent	referent
>=1	2.9 (2 to 4)	<0.0001	2.9 (2 to 4)	0.0001	2.8 (2 to 4)	0.0001
Marital Status						
Married/Living with partner	referent	referent	referent	referent	referent	referent
Widowed/Divorced/Separated	−0.5 (−2 to 1)	0.387	−0.5 (−2 to 1)	0.371	−0.4 (−1 to 1)	0.425
Alcohol Consumption						
<12 drinks/yr			referent	referent	referent	referent
>12 drinks/yr			1 (0.2 to 2)	0.02	1 (0.1 to 2)	0.034
HTN						
No					referent	referent
Yes					−1 (−2 to 0.1)	0.078
DM						
No					referent	referent
Yes					−1.7 (−2 to −1)	<0.0001
Stroke						
No					referent	referent
Yes					−0.6 (−2 to 1)	0.444
CAD						
No					referent	referent
Yes					−1.8 (−4 to 0.1)	0.065

*Unadjusted.† Adjusted for age, gender, ethnicity, education, PIR, and marital status. ‡ Adjusted for age, gender, ethnicity, education, PIR, marital status, and alcohol consumption. § Adjusted for age, gender, ethnicity, education, PIR, marital status, alcohol consumption, HTN, DM, stroke, and CAD. ¶ Weighted β and 95% Confidence Interval.

Table S5. Mn-Urine univariate analyses of the association between z-score and covariates (*n* = 950).

Variables	Score Mean (SE)*	*F* value	*P*-value
Gender		94.11	<0.0001
Male	0.13 (0.05)		
Female	0.32 (0.05)		
Age (year)	-	4.58**	<0.0001
Race/ethnicity		29.4	<0.0001
Hispanic	−0.31 (0.05)		
NH White	0.33 (0.05)		
NH Black	−0.07 (0.04)		
Other race	0.03 (0.1)		
Education		64	<0.0001
<High School	−0.37 (0.05)		
High School	0.02 (0.06)		
>High School	0.46 (0.05)		
PIR		37.18	<0.0001
<=0.99	−0.28 (0.06)		
>=1	0.31 (0.05)		
Marital Status		23.86	<0.0001
Married/Living with partner	0.29 (0.05)		
Widowed/Divorced/Separated	0.09 (0.06)		
Alcohol Consumption		21.89	<0.0001
>12 drinks/yr	0.31 (0.05)		
<12 drinks/yr	0.04 (0.05)		
HTN		38.67	<0.0001
Yes	0.13 (0.06)		
No	0.37 (0.06)		
DM		30.96	<0.0001
Yes	−0.01 (0.05)		
No	0.29 (0.05)		
CAD		38.69	<0.0001
Yes	−0.23 (0.07)		
No	0.28 (0.04)		
Stroke		31.63	<0.0001
Yes	−0.11 (0.2)		
No	0.24 (0.04)		

*Weighted mean and SE; **t-score

Table S6. Mn-Urine levels (continuous) in relation to CERAD (delayed).

	Model 1 (*n* = 2068)*		Model 2 (*n* = 1772)†		Model 3 (*n* = 1744)‡		Model 4 (*n* = 1650)§	
Variables	*β* (95% CI)¶	*P*-value	*β* (95% CI)¶	*P*-value	*β* (95% CI)¶	*P*-value	*β* (95% CI)¶	*P*-value
Mn	0.4 (−1 to 2)	0.54	0.1 (−2 to 0.20)	0.96	0.1 (−2 to 2)	0.95	−3 (−5 to 0.1)	0.06
Gender								
Male			referent	referent	referent	referent	referent	referent
Female			8 (7 to 10)	<0.0001	8 (7 to 10)	<0.0001	8 (5 to 10)	<0.0001
Age (year)			−1 (−2 to −1)	<0.0001	−13 (−16 to −1)	<0.0001	−1.2 (−2 to −0.9)	<0.0001
Race/Ethnicity								
NH White			referent	referent	referent	referent	referent	referent
Hispanic			−10 (−15 to −4)	0.001	−10 (−15 to −4)	0.002	−10 (−16 to −5)	0.001
NH Black			−4 (−7 to −0.5)	0.03	−3 (−7 to −0.5)	0.02	−4 (−7 to −0.3)	0.03
Other Race			−0.6 (−7 to 6)	0.84	−0.7 (−6 to 5)	0.81	−1 (−7 to 5)	0.7
Education								
>High School			referent	referent	referent	referent	referent	referent
High School			−7 (−11 to −2)	0.01	−6 (−11 to −2)	0.01	−6 (−10 to −2)	0.01
<High School			−7 (−13 to −1)	0.02	−7 (−13 to −1)	0.02	−7 (−10 to −1)	0.03
PIR								
<=0.99			referent	referent	referent	referent	referent	referent
>=1			4 (−2 to 9)	0.18	3 (−2 to 9)	0.25	3 (−3 to 9)	0.28
Marital Status								
Married/Living with partner			referent	referent	referent	referent	referent	referent
Widowed/Divorced/Separated			−2 (−6 to 3)	0.46	−2 (−6 to 2)	0.39	−1 (−6 to 3)	0.49
Alcohol Consumption								
<12 drinks/yr					referent	referent	referent	referent
>12 drinks/yr					1 (−3 to 5)	0.47	2 (−3 to 6)	0.48
HTN								
No							referent	referent
Yes							−4 (−8 to 1)	0.12
DM								
No							referent	referent
Yes							1 (−2 to 5)	0.52
Stroke								

No							referent	referent
Yes							−9 (−10 to −3)	0.01
CAD								
No							referent	referent
Yes							−5 (−20 to 10)	0.4

*Unadjusted.† Adjusted for age, gender, ethnicity, education, PIR, and marital status. ‡ Adjusted for age, gender, ethnicity, education, PIR, marital status, and alcohol consumption. § Adjusted for age, gender, ethnicity, education, PIR, marital status, alcohol consumption, HTN, DM, stroke, and CAD. ¶ Weighted β and 95% Confidence Interval.

Table S7. Mn-Urine levels (continuous) in relation to AF.

	Model 1 (*n* = 2068)*		Model 2 (*n* = 1772)†		Model 3 (*n* = 1744)‡		Model 4 (*n* = 1650)§	
Variables	*β* (95% CI)¶	*P*-value	*β* (95% CI)¶	*P*-value	*β* (95% CI)¶	*P*-value	*β* (95% CI)¶	*P*-value
Mn	1 (−3 to 6)	0.48	−0.2 (−6 to 6)	0.94	−0.3 (−6 to 5)	0.9	−4 (−20 to 10)	0.51
Gender								
Male			referent	referent	referent	referent	referent	referent
Female			−6 (−16 to 4)	0.24	−4 (−14 to 6)	0.42	−2 (−10 to 10)	0.66
Age (year)			−3 (−4 to −2)	<0.0001	−3 (−4 to −2)	<0.0001	−3 (−4 to −2)	<0.0001
Race/Ethnicity								
NH White			referent	referent	referent	referent	referent	referent
Hispanic			−30 (−40 to −20)	<0.0001	−30 (−40 to −20)	<0.0001	−30 (−40 to −20)	<0.0001
NH Black			−40 (−50 to −20)	<0.0001	−30 (−50 to −20)	<0.0001	−30 (−50 to −20)	<0.0001
Other Race			−30 (−40 to −20)	<0.0001	−30 (−40 to −20)	<0.0001	−30 (−40 to −20)	0.0001
Education								
>High School			referent	referent	referent	referent	referent	referent
High School			−30 (−40 to −20)	<0.0001	−30 (−40 to −20)	<0.0001	−30 (−40 to −20)	<0.0001
<High School			−30 (−40 to −20)	<0.0001	−30 (−40 to −20)	<0.0001	−30 (−40 to −10)	0.0002
PIR								
<=0.99			referent	referent	referent	referent	referent	referent
>=1			10 (−3 to 30)	0.12	10 (−4 to 30)	0.15	10 (−6 to 20)	0.24
Marital Status								
Married/Living with partner			referent	referent	referent	referent	referent	referent
Widowed/Divorced/Separated			4 (−8 to 20)	0.48	4 (−8 to 20)	0.51	4 (−9 to 20)	0.58
Alcohol Consumption								

<12 drinks/yr					referent	referent	referent	referent
>12 drinks/yr					10 (−1 to 30)	0.07	10 (−1 to 30)	0.06
HTN								
No							referent	referent
Yes							−10 (−20 to −2)	0.02
DM								
No							referent	referent
Yes							−7 (−20 to 5)	0.23
Stroke								
No							referent	referent
Yes							−3 (−20 to 10)	0.74
CAD								
No							referent	referent
Yes							−20 (−40 to 10)	0.23

*Unadjusted.† Adjusted for age, gender, ethnicity, education, PIR, and marital status. ‡ Adjusted for age, gender, ethnicity, education, PIR, marital status, and alcohol consumption. § Adjusted for age, gender, ethnicity, education, PIR, marital status, alcohol consumption, HTN, DM, stroke, and CAD. ¶ Weighted β and 95% Confidence Interval.

Table S8. Mn-Urine levels (continuous) in relation to cognitive function.

	Model 1 (*n* = 2068)*		Model 2 (*n* = 1772)†		Model 3 (*n* = 1744)‡		Model 4 (*n* = 1650)§	
Variables	*β* (95% CI)¶	*P*-value	*β* (95% CI)¶	*P*-value	*β* (95% CI)¶	*P*-value	*β* (95% CI)¶	*P*-value
Mn	−0.3 (−1 to 0.3)	0.3073	−0.6 (−1 to 0.3)	0.1916	−0.6 (−1 to 0.0)	0.1298	−1 (−2 to 0.3)	0.0804
Gender								
Male			referent	referent	referent	referent	referent	referent
Female			3 (2 to 4)	<0.0001	3 (02 to 4)	<0.0001	3 (2 to 4)	<0.0001
Age (year)			−1 (−1 to −0.5)	<0.0001	−0.5 (−1 to −0.4)	<0.0001	−0.5 (−1 to −0.4)	<0.0001
Race/Ethnicity								
NH White			referent	referent	referent	referent	referent	referent
Hispanic			−5 (−6 to −4)	<0.0001	−5 (−6 to −4)	<0.0001	−5 (−7 to −4)	<0.0001
NH Black			−4 (−5 to −2)	<0.0001	−3 (−5 to −2)	<0.0001	−3 (−5 to −2)	<0.0001
Other Race			−3 (−5 to −1)	0.0132	−2 (−4 to 0.4)	0.0167	−2 (−4 to 0.4)	0.0222
Education								
>High School			referent	referent	referent	referent	referent	referent

High School	−5 (−7 to −4)	<0.0001	−5 (−5 to −2)	<0.0001	−5 (−6 to −3)	<0.0001
<High School	−4 (−5 to −3)	<0.0001	−4 (−5 to −2)	<0.0001	−3 (−5 to −2)	<0.0001
PIR						
<=0.99	referent	referent	referent	referent	referent	referent
>=1	2 (0.4 to 4)	0.0172	2 (0.2 to 4)	0.0334	2 (0.4 to 4)	0.0466
Marital Status						
Married/Living with partner	referent	referent	referent	referent	referent	referent
Widowed/Divorced/Separated	−0.7 (−2 to 0.8)	0.3549	−0.8 (−2 to 0.6)	0.2856	−0.7 (−2 to 0.6)	0.2821
Never married	−0.6 (−3 to 2)	0.615	−0.6 (−3 to 2)	0.6064	−0.7 (−3 to 2)	0.5302
Alcohol Consumption						
<12 drinks/yr			referent	referent	referent	referent
>12 drinks/yr			2 (0.1 to 3)	0.0363	1 (0.003 to 3)	0.0504
HTN						
No					referent	referent
Yes					−1 (−3 to −0.1)	0.0328
DM						
No					referent	referent
Yes					−1 (−2 to −0.2)	0.0186
Stroke						
No					referent	referent
Yes					−2 (−4 to −0.5)	0.1277
CAD						
No					referent	referent
Yes					−2 (−6 to −02)	0.3299

**Unadjusted.† Adjusted for age, gender, ethnicity, education, PIR, and marital status. ‡ Adjusted for age, gender, ethnicity, education, PIR, marital status, and alcohol consumption. § Adjusted for age, gender, ethnicity, education, PIR, marital status, alcohol consumption, HTN, DM, stroke, and CAD. ¶ Weighted β and 95% Confidence Interval.
